# Characterization of GPVI- or GPVI-CD39-Coated Nanoparticles and Their Impact on In Vitro Thrombus Formation

**DOI:** 10.3390/ijms23010011

**Published:** 2021-12-21

**Authors:** Jeremy A. Nestele, Anne-Katrin Rohlfing, Valerie Dicenta, Alexander Bild, Daniela Eißler, Frederic Emschermann, Marcel Kremser, Konstantin Krutzke, Tilman E. Schäffer, Oliver Borst, Moran Levi, Netanel Korin, Meinrad Paul Gawaz

**Affiliations:** 1 Department of Cardiology and Angiology, University of Tübingen, 72076 Tubingen, Germany; Jeremy.Nestele@med.uni-tuebingen.de (J.A.N.); Anne-Katrin.Rohlfing@med.uni-tuebingen.de (A.-K.R.); Valerie.Dicenta@med.uni-tuebingen.de (V.D.); Alexander.Bild@med.uni-tuebingen.de (A.B.); Daniela.Eissler@med.uni-tuebingen.de (D.E.); Frederic.Emschermann@med.uni-tuebingen.de (F.E.); Marcel.Kremser@med.uni-tuebingen.de (M.K.); Oliver.Borst@med.uni-tuebingen.de (O.B.); 2 DFG Heisenberg Group Thrombocardiology, University of Tübingen, 72076 Tubingen, Germany; 3 Institute for Applied Physics, University of Tübingen, 72076 Tubingen, Germany; konstantin.krutzke@uni-tuebingen.de (K.K.); tilman.schaeffer@uni-tuebingen.de (T.E.S.); 4 Department of Biomedical Engineering, Technion, Israel Institute of Technology, Haifa 3200003, Israel; smoranle@campus.technion.ac.il (M.L.); korin@bm.technion.ac.il (N.K.)

**Keywords:** nanoparticles, hemostasis, antithrombotic therapy, platelets

## Abstract

Traditional antithrombotic agents commonly share a therapy-limiting side effect, as they increase the overall systemic bleeding risk. A novel approach for targeted antithrombotic therapy is nanoparticles. In other therapeutic fields, nanoparticles have enabled site-specific delivery with low levels of toxicity and side effects. Here, we paired nanotechnology with an established dimeric glycoprotein VI-Fc (GPVI-Fc) and a GPVI-CD39 fusion protein, thereby combining site-specific delivery and new antithrombotic drugs. Poly(lactic-co-glycolic acid) (PLGA) nanoparticles, NP-BSA, NP-GPVI and NP-GPVI-CD39 were characterized through electron microscopy, atomic force measurements and flow cytometry. Light transmission aggregometry enabled analysis of platelet aggregation. Thrombus formation was observed through flow chamber experiments. NP-GPVI and NP-GPVI-CD39 displayed a characteristic surface coating pattern. Fluorescence properties were identical amongst all samples. NP-GPVI and NP-GPVI-CD39 significantly impaired platelet aggregation. Thrombus formation was significantly impaired by NP-GPVI and was particularly impaired by NP-GPVI-CD39. The receptor-coated nanoparticles NP-GPVI and the bifunctional molecule NP-GPVI-CD39 demonstrated significant inhibition of in vitro thrombus formation. Consequently, the nanoparticle-mediated antithrombotic effect of GPVI-Fc, as well as GPVI-CD39, and an additive impact of CD39 was confirmed. In conclusion, NP-GPVI and NP-GPVI-CD39 may serve as a promising foundation for a novel therapeutic approach regarding targeted antithrombotic therapy.

## 1. Introduction

According to the World Health Organization, approximately 30% of all deaths are linked to cardiovascular diseases (CVD), including, e.g., coronary heart disease and cerebrovascular disease, and, thereby, represent the predominant cause of death worldwide [1]. Atherothrombosis and vascular ischemia form core pathophysiological features of CVD with platelets playing a key role in thrombogenesis [2,3]. Presently, antithrombotic agents have become a fundamental pillar in the prevention of CVD [4]. Classical platelet inhibiting agents are cyclooxygenase inhibitors and adenosine diphosphate (ADP) receptor blockers. However, due to their systemic mode of action and the inhibition of platelet function, they increase the overall bleeding risk, thus generating a major limiting factor in traditional antithrombotic therapy [5].

New antithrombotic agents are currently under investigation and are paramount to reduce systemic side effects [6]. Among those, Revacept^®^, a recombinant dimeric glycoprotein (GP) VI-Fc, has proven to inhibit thrombus formation without altering bleeding time [7,8,9,10,11]. The collagen receptor GPVI subunit of Revacept^®^ can bind exposed, subendothelial collagen, which is a trigger for arterial atherosclerotic plaque formation. By locally binding to exposed collagen, Revacept^®^ occupies platelet binding sites, thus preventing platelet binding, activation and subsequent thrombus formation. By synthetically fusing GPVI-Fc to ectonucleotidase cluster of differentiation 39 (CD39), an adenosine diphosphate (ADP)-degrading enzyme [12], Degen et al. created a bifunctional molecule (GPVI-CD39) that boosts the antithrombotic effect at sites of vascular lesions in in vitro and in animal models. ADP is a known platelet activator and is also released by activated platelets themselves. The enzymatic activity of CD39 obstructs the multiplier effect of ADP release from activated platelets by locally degrading and thereby inactivating free ADP into inactive adenosine monophosphate (AMP) [13].

Just as antithrombotic agents advanced, nanotechnology has revolutionized several scientific fields. Nanomedicine is emerging as a whole new field of expertise and may offer a new treatment paradigm [14]. Nanoparticles (NP), as vectors of nanotechnology, promise a novel method of targeted therapy with advantages in pharmacodynamics and kinetics, biodegradability, immunogenicity and toxicity [15]. Since 2009, 15 nanotherapeutics have gained market approval [16]. Especially in oncology, nanoparticles are used to enhance the efficiency and lower the systemic toxicity of chemotherapeutics [17,18]. In contrast, attempts to use nanotherapeutics for the treatment of CVD have been rare. For example, Korin et al. developed flow-dependent, tissue plasminogen activator (t-PA)-coated nanoparticle aggregates. The flow acceleration and high shear rates at the locations of a vascular stenosis lead to localized disintegration of the aggregates. The resulting confined release of t-PA subsequently induced thrombolysis [19]. In addition, NP-GPVI has been successfully studied for targeted delivery to sites of vessel injury in vitro and in vivo as well as in high-risk aneurysms [20,21].

Here, we wanted to understand the application possibilities of nanoparticles in the prevention of thrombus formation, particularly in combination with the enhanced properties of the GPVI-CD39 fusion protein. Therefore, we generated GPVI-Fc- and GPVI-CD39-coated nanoparticles. The produced nanoparticles were characterized in regard to size, receptor load and fluorescence properties. We hypothesized that these nanoparticles would bind and block collagen and, therefore, would prevent platelet binding and activation, as well as aggregation. The enzymatic activity of the additional CD39 subunit within the NP-GPVI-CD39 construct would degrade and, thus, inactivate ADP. This would locally inhibit ADP-mediated platelet activation and thereby prevent an activation cascade promoting thrombus formation. We demonstrated that NP-GPVI and NP-GPVI-CD39 effectively adhered to collagen fibers under both static and dynamic conditions, using NP-BSA as an inert control. As expected, receptor-coated nanoparticles inhibited in vitro platelet aggregation. Furthermore, NP-GPVI and NP-GPVI-CD39 interfered with in vitro thrombus formation under dynamic conditions. As hypothesized, CD39 had a significant additive effect on the prevention of thrombus formation compared to GPVI-Fc alone.

## 2. Results

### 2.1. Nanoparticle Generation and Charaterization

**Figure 1:**
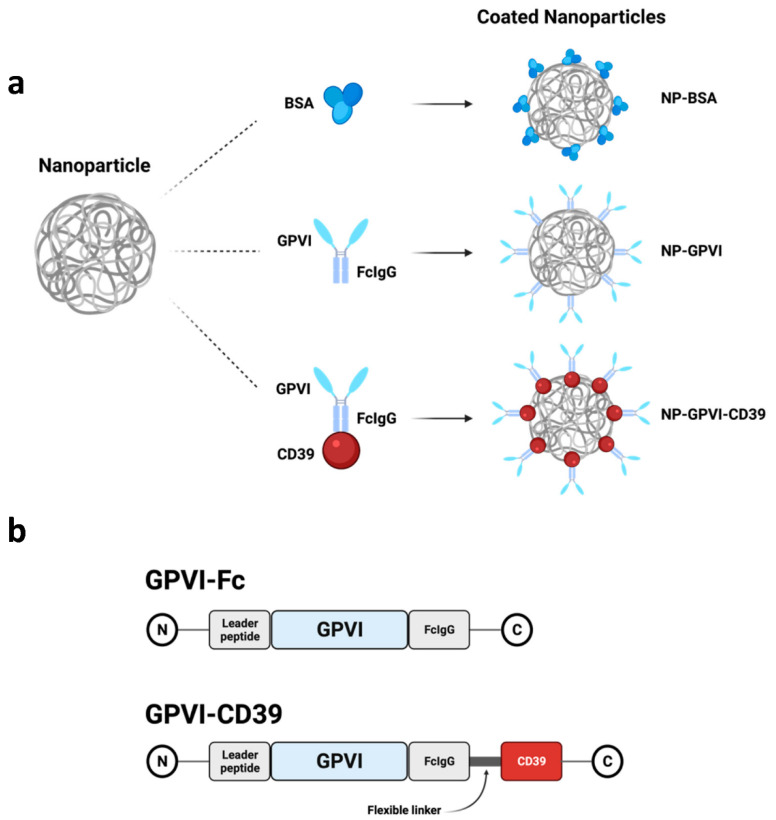
Schematic presentation of coated nanoparticles and their functional receptors GPVI-Fc and GPVI-CD39. (**a**) poly(lactic-co-glycolic acid)(PLGA)-nanoparticles with their respective coating agents BSA, GPVI-Fc and GPVI-CD39. (**b**) Molecular architecture of the functional receptor proteins GPVI-Fc and GPVI-CD39. Created with BioRender.com; accessed on 17 June 2021).

Poly(lactic-co-glycolic acid) (PLGA) nanoparticles measuring 200 nm and containing fluorescent dye (nile red) were produced and coated with bovine serum albumin (BSA), GPVI-Fc (Revacept^®^) or GPVI-CD39 as described previously (see methods and Levi et al.) (Figure 1) [20]. Z-average size and zeta-potential were determined using a dynamic light scattering (DLS) instrument (NP-BSA—diameter: 207 ± 95 nm, Zeta: −21.7 ± 6.3 mV; NP-GPVI—diameter: 261 ± 105 nm, Zeta: −24.1 ± 4.2 mV; NP-GPVI-CD39—diameter 314 ± 78 nm, Zeta −17± 4.2 mV). Average coating densities on the NPs were estimated via a standard Bradford assay (NP-BSA—17.000, NP-GPVI—1.500 and NP-GPVI-CD39—1.000 molecules per particle).

**Figure 2:**
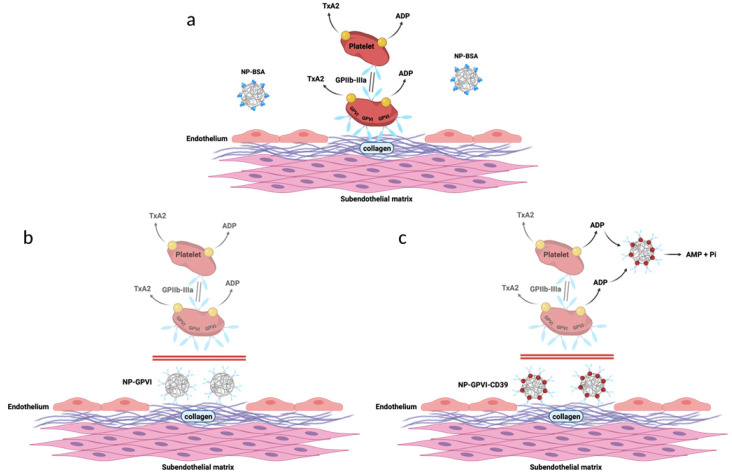
Schematic presentation of the hypothesized nanoparticle-induced modification of the hemostatic response following endothelial damage. Nanoparticle-specific mode of action at sites of endothelial damage. Due to the dimeric collagen receptor GPVI-Fc, both NP-GPVI (**b**) and NP-GPVI-CD39 (**c**) adhere to subendothelial collagen, thus competitively inhibiting platelet adhesion and collagen-induced platelet activation. Additionally, NP-GPVI-CD39 (**c**) degrades platelet-released ADP through ectonucleotidase CD39, thereby bifunctionally impairing platelet activation. (**a**) NP-BSA does not affect platelet adhesion. Created with BioRender.com; accessed on 18 July 2021).

As depicted in Figure 2, both NP-GPVI and NP-GPVI-CD39 hypothetically occupy collagen binding sites through dimeric GPVI-Fc and should thereby inhibit thrombus formation. Furthermore, the enzymatic CD39 subunit of NP-GPVI-CD39 degrades ADP, a known platelet activator (Figure 2c) [22]. Thus, NP-GPVI-CD39 serves as a bifunctional antithrombotic agent, inhibiting both platelet binding and activation. BSA does not interfere with the hemostatic process and, thus, NP-BSA was used as a control (Figure 2a). To exclude general cytotoxicity, we performed a cell viability assay (see supplemental Figure S1) and found no negative effect of the three NP constructs on cells.

**Figure 3:**
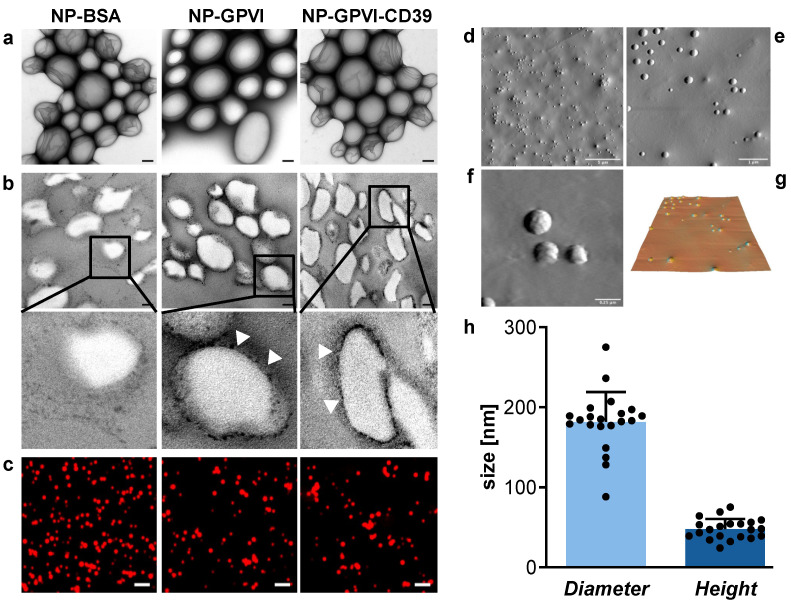
Morphological characterization of the nanoparticles (**a**,**b**) Electron microscopic display of nanoparticles, (**a**) negative staining, scale 100 nm, (**b**) ultra-thin sectioning, scale 100 nm, illustrates the coating differences amongst the nanoparticles. NP-GPVI and NP-GPVI-CD39 display their functional coating proteins as a distinctive surface coating pattern (arrowheads). (**c**) confocal laser scanning microscopy of fluorescent nanoparticles, scale 2 μm. (**d**–**f**): Topographical portrayal of NP-BSA through atomic force microscopy (AFM) in increasing magnification; (**d**) scale bar = 5 µm, (**e**) scale bar = 1 µm, (**f**) scale bar = 0.25 µm, (**g**) three-dimensional transformation of (**e**), (**h**) Quantitative analysis of the morphology parameters diameter and height, based on AFM measurements. Mean ± SD, *n* = 21.

In order to assess the morphological properties of the nanoparticles used, electron microscopic assays, as well as atomic force measurements, were conducted. Ultrathin sectioning revealed a characteristic surface coating of both NP-GPVI and NP-GPVI-CD39, represented by a halo of electron-dense material surrounding the NP (Figure 3, arrow heads). Atomic force measurements (AFM) were used to determine the dimensional parameters of NP-BSA (Figure 3d–g). The mean nanoparticle diameter over all samples was 182 ± 37 nm with an average height of 48 ± 13 nm (Figure 3h). The diameter measurements coincided with the expected size of the nanoparticles produced by the applied method [20]. The low height could be due to the fixation process needed for the AFM or due to the NPs sinking into the collagen-containing matrix, which was needed for NP adherence in the first place.

**Figure 4:**
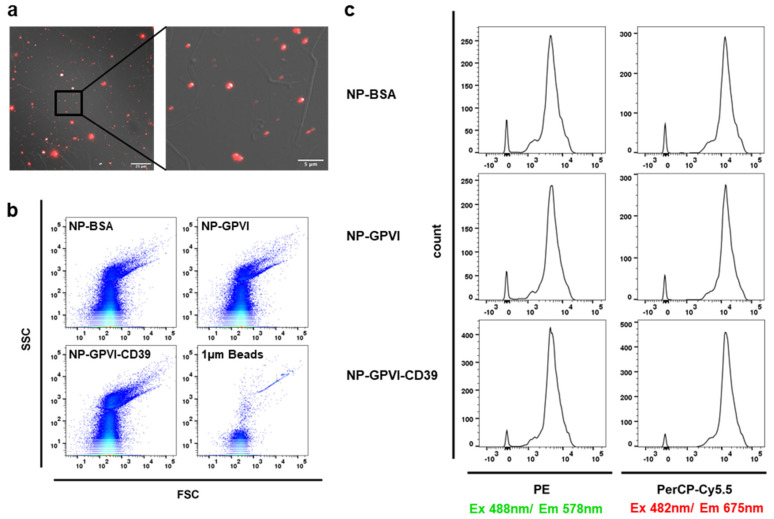
Fluorescence properties of the nanoparticles (**a**) Merged differential interference contrast (DIC) and fluorescence microscopy image of the nanoparticles (nile red) after adhesion to collagen-coated cover slips. 100× magnification, scale bar = 25 µm. (**b**) Flow cytometric detection of the nanoparticles, demonstrating the particle distribution of all three nanoparticle samples, as well as a sample containing 1 µm beads, in both FSC and SSC detection channels. (**c**) Histograms displaying the fluorescence intensities of the nanoparticle samples in flow cytometric analysis in both phycoerythrin (PE) and peridinin chlorophyll protein (PerCP) fluorochrome detection channels after 488 nm excitation.

The incorporation of the fluorescent dye nile red within the nanoparticles enabled their detection by fluorescence-based techniques (Figure 4). The nanoparticles were clearly visible by high-resolution fluorescent microscopy (Figure 4a). Flow cytometry analysis of the emmitted nile red fluorescence using two detection channels (phycoerythrin—PE and peridinin chlorophyll protein—PerCP) revealed no differences in the fluorescent intensity of the three different nanoparticle samples, allowing the use of fluorescence-based assays (Figure 4c). In addition, the nanoparticle diameter was determined by flow cytometric measurements to be smaller than 1 µm polystyrene beads (Figure 4b). The majority of the nanoparticle distribution pattern was situated to the left of the 1 µm polystyrene bead sample on the FSC scale, demonstrating a size smaller the 1 µm. Reconfirming the aspired size of 200 nm. The particle granularity, measured by the SSC detection channel, was also comparable between the three nanoparticle samples.

### 2.2. GPVI-Fc- and GPVI-CD39-Coated Nanoparticles Effectively Adhere to Collagen under Both Static and Dynamic Conditions

**Figure 5:**
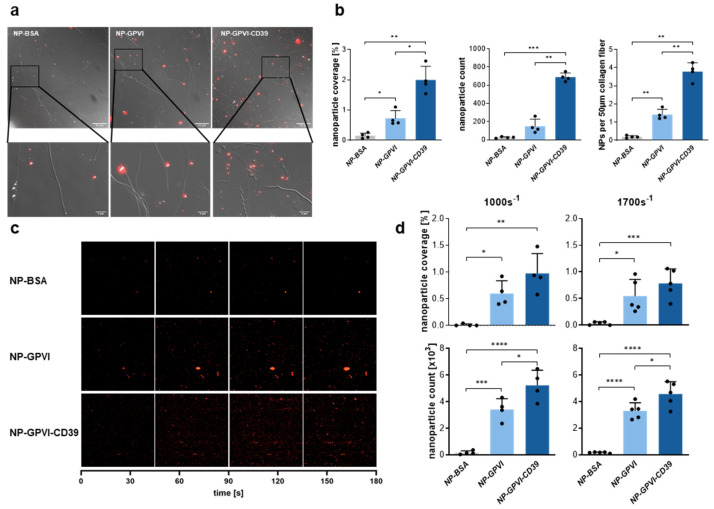
Collagen receptor GPVI-Fc- and GPVI-CD39-coated nanoparticles effectively bind to collagen fibers under both static and dynamic conditions. (**a**) Merged DIC and fluorescence microscopy images of nanoparticles adhered onto collagen coated cover slips after 1 h incubation at 37 °C. 100× magnification. Scale bar: upper row, 25 µm; lower row, 5 µm. (**b**) Statistical analysis of nanoparticle coverage, total nanoparticle count and nanoparticle count per 50 µm of collagen fiber. Mean ± SD; *n* = 4; ordinary one-way ANOVA; * *p* ≤ 0.05, ** *p* ≤ 0.01, *** *p* ≤ 0.001. (**c**) Representative time-series images depicting nanoparticles adhering to collagen-covered cover slips in flow chamber experiments at HS (1700 s^−1^). 20× magnification. (**d**) Statistical analysis of nanoparticle coverage and total nanoparticle count per shear rate of NP-GPVI, NP-GPVI-CD39 and NP-BSA. Mean ± SD; 1000 s^−1^: *n* = 4, 1700 s^−1^: *n* = 5; ordinary one-way ANOVA; * *p* ≤ 0.05, ** *p* ≤ 0.01, *** *p* ≤ 0.001, **** *p* < 0.0001.

To study the ability of the coated nanoparticles to adhere to collagen fibers, static adhesion experiments were performed. The adhesion of nanoparticles to collagen-coated cover slips was visualized in merged differential interference contrast (DIC) and fluorescent (nile red) images taken with a 100× objective (Figure 5a). An augmented collagen-binding capacity of both NP-GPVI and NP-GPVI-CD39 compared to NP-BSA was observed in these images (Figure 5a,b). A subsequent statistical analysis confirmed the enhanced static collagen fiber binding capacity of both NP-GPVI and NP-GPVI-CD39 compared to NP-BSA (Figure 5b). The average number of adhesive nanoparticles per 50 µm of collagen fiber was significantly increased for both NP-GPVI (7.5-fold; 1.42 ± 0.29) and NP-GPVI-CD39 (19.9-fold; 3.78 ± 0.49) compared to NP-BSA (0.19 ± 0.07). In addition, nanoparticle coverage and the total amount of detected nanoparticles within a defined area were significantly elevated for both NP-GPVI (coverage: 0.73 ± 0.25%; count: 149 ± 77.8 × 10^3^) and NP-GPVI-CD39 (coverage: 1.99 ± 0.45%; count: 688.9 ± 46.79 × 10^3^), compared to NP-BSA (coverage: 0.15 ± 0.09%; count: 26.8 ± 7.89 × 10^3^).

Using our established flow chamber model, we also analyzed the collagen-binding capacity of nanoparticles under dynamic flow conditions. Low shear (LS; 1000 s^−1^) and high shear (HS; 1700 s^−1^) rates were applied and compared in this experimental setup (Figure 5c,d). At both shear rates, NP-GPVI as well as NP-GPVI-CD39 demonstrated a significantly enhanced collagen fiber binding capacity compared to NP-BSA particles (Figure 5d). In detail, compared to NP-BSA (LS: 0.01 ± 0.02%; HS: 0.032 ± 0.030%), the nanoparticle coverage of a defined area was significantly increased in NP-GPVI (LS: 60-fold, 0.6 ± 0.24%; HS: 17-fold, 0.55 ± 0.31%) and NP-GPVI-CD39 (LS: 98-fold, 0.98 ± 0.37%; HS: 25-fold, 0.78 ± 0.27%) samples. Furthermore, the total nanoparticle count within a defined area was significantly higher for NP-GPVI (LS: 19-fold, 3.24 ± 0.81; HS: 19-fold, 3.31 ± 0.61) and NP-GPVI-CD39 (LS: 30-fold, 5.23 ± 1.14; HS: 26-fold, 4.57 ± 0.93). Thus, the generated nanoparticles demonstrated a promising collagen-binding capacity. The next step was to test their ability to inhibit in vitro aggregation and thrombus formation.

### 2.3. Receptor-Coated Nanoparticles Inhibit In Vitro Platelet Aggregation

**Figure 6:**
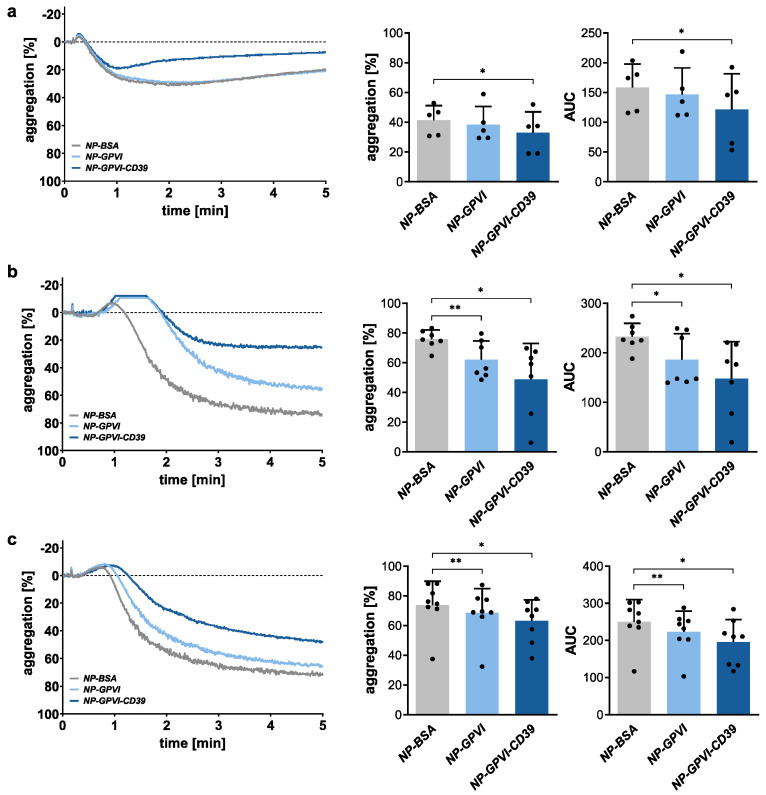
Receptor-coated nanoparticles inhibit platelet aggregation in light transmission aggregometry. **Left**: Representative light transmission aggregation curves of PRP activated by the three different platelet activators, ADP (**a**), collagen (**b**) and CRP (**c**). **Right**: Statistical analysis of maximum aggregation and AUC. Mean ± SD; ADP: *n* = 5, Collagen: *n* = 7, CRP: *n* = 8; one-way ANOVA; * *p* ≤ 0.05, ** *p* ≤ 0.01.

To demonstrate the ability of the generated nanoparticles to influence platelet aggregation in vitro, we performed light transmission aggregometry assays (Figure 6). These experiments were performed with platelet-rich plasma (PRP) using an establish protocol on a CHRONO-LOG aggregometer 490-X (Havertown, PA, USA). Three different platelet agonists, adenosine diphosphate (ADP, 5 µM), collagen (20 µM) and collagen-related peptide (CRP, 0.125 µg/mL), were used to activate platelets in this experimental setup. Each nanoparticle was pre-incubated for 30 min at 37 °C with the individual agonist. Pre-treatment was performed to evaluate the effect of the three different NPs on the tested agonists. Especially, the impact of the enzymatic activity of CD39 (NP-GPVI-CD39) on ADP degradation and, thereby, deactivation to AMP (Figure 2c). Indeed, the bifunctional NP-GPVI-CD39 significantly inhibited ADP-dependent aggregation in vitro compared to NP-BSA and NP-GPVI (Figure 6a). The maximum aggregation (MA) decreased by 20% (32.98 ± 14.05) and the area under the curve (AUC) decreased by 23% (121.6 ± 60.1) compared to NP-BSA (MA: 41.39 ± 9.82; AUC: 158.7 ± 39.36). NP-GPVI alone did not influence this activation pathway (MA: 38.47 ± 12.22%; AUC: 147.1 ± 44.22). In contrast, collagen activation was significantly compromised in NP-GPVI and NP-GPVI-CD39 samples when compared to NP-BSA (Figure 6b). NP-GPVI and NP-GPVI-CD39 reduced MA by 18% (62.12 ± 12.58%) and 36% (48.91 ± 24.03%), respectively, as well as AUC by 20% (186.1 ± 52.75) and 36% (147.8 ± 74.93), respectively, in comparison to NP-BSA (75.87 ± 6.16%; AUC: 232.6 ± 27.03). Comparable results were observed under CRP activation, as both NP-GPVI and NP-GPVI-CD39 significantly reduced MA by 7% (68.64 ± 16.25%) and 14% (63.23 ± 14.11%) as well as AUC by 10% (223.0 ± 56.04) and 22% (195.4 ± 60.81), respectively, in comparison to NP-BSA (MA: 73.82 ± 16.08%; AUC: 250.2 ± 59.96) (Figure 6c). Therefore, the created functional NPs had an impact on in vitro platelet aggregation. Moreover, the enzymatic subunit CD39 had a significant additive effect.

### 2.4. Receptor-Coated Nanoparticles Interfere with In Vitro Thrombus Formation under Dynamic Conditions

**Figure 7:**
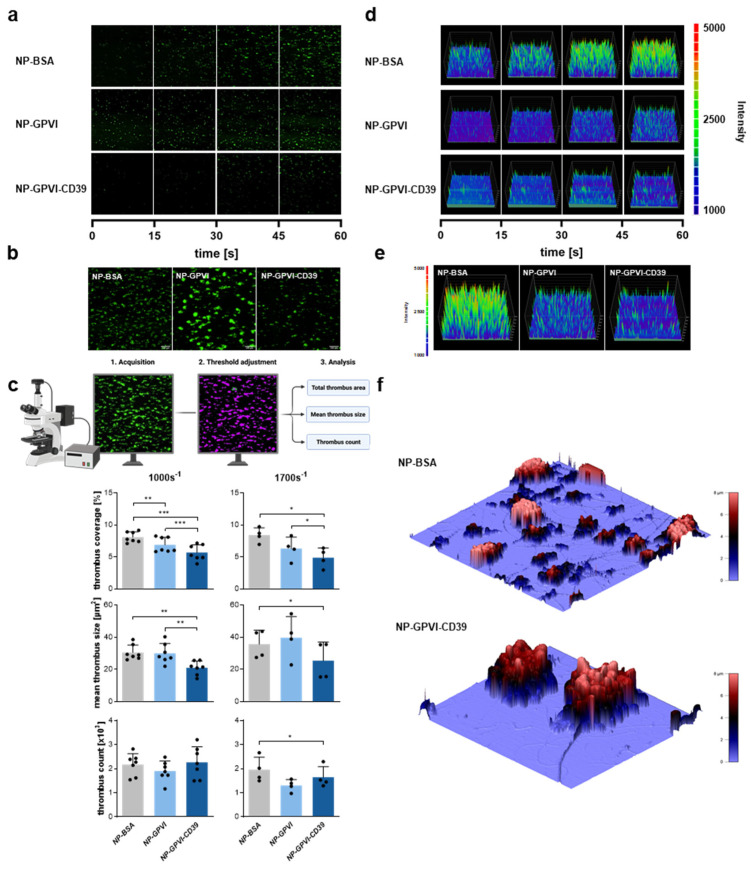
Interference of receptor-coated nanoparticles with thrombus formation under dynamic conditions. (**a**) Representative time-series images of flow chamber experiments depicting thrombus formation on collagen-covered cover slips after pre-incubation with nanoparticles, scale bar = 100 µm. (**b**) Representative fluorescence microscopic images of thrombus formation after perfusion, scale bar = 100 µm. (**c**) Schematic presentation of the analytical process of the conducted perfusion experiments. Created with BioRender.com; accessed on 17 June 2021). Statistical analysis of thrombus coverage, mean thrombus size and thrombus count at both low (1000 s^−1^) and high (1700 s^−1^) shear rates. Mean ± SD; 1000 s^−1^: *n* = 7, 1700 s^−1^; *n* = 4; one-way ANOVA, * *p* ≤ 0.05, ** *p* ≤ 0.01, *** *p* ≤ 0.001. (**d**) Time series of intensity surface plots representing the acquired flow chamber data. (**e**) Representative intensity surface plots of the completed perfusion experiments. (**f**) Topographical display of thrombus-covered cover slips after completed perfusion at a high shear rate (1700 s^−1^), produced by scanning ion-conductance microscopy (SICM).

To add biological significance, perfusion experiments at low and high shear rates were conducted to evaluate the influence of the nanoparticles on in vitro thrombus formation (Figure 7). In this experimental setup, the NP-coated cover slips were used, with collagen as an activating substrate for whole blood. Time-series images depict the thrombus formation over time on the differently coated cover slips (Figure 7a) and already display decreased thrombus formation in samples containing functional NPs (NP-GPVI and NP-GPVI-CD39) compared to controls (NP-BSA). Statistical analysis of fluorescent images taken after completed perfusion and rinsing of the samples revealed significantly reduced thrombus coverages, mean thrombus sizes and thrombus count in NP-GPVI and NP-GPVI-CD39 compared to NP-BSA at low and high shear stress (Figure 7c). In detail, NP-GPVI significantly reduced thrombus coverage by 15% (6.89 ± 1.16%) and NP-GPVI-CD39 reduced thrombus coverage by 30% (5.67 ± 1.23%) at LS, compared to NP-BSA (8.1 ± 0.83%). At high shear stress, NP-GPVI-CD39 significantly decreased thrombus coverage by 42% (4.87 ± 1.52%), when compared to NP-BSA (8.4 ± 1.15%). NP-GPVI-CD39 also significantly reduced thrombus coverage by 18% at LS and 23% at HS, when compared to NP-GPVI (LS: 6.89 ± 1.16%; HS: 6.29 ± 1.78%).

Planimetric analysis of mean thrombus size showed a significantly smaller mean thrombus size for NP-GPVI-CD39 at both LS (20.86 ± 6.19 µm^2^) and HS (25.36 ± 11.59 µm^2^) in comparison to NP-BSA (LS: 30.47 ± 4.71 µm^2^; HS: 35.64 ± 8.75 µm^2^). When compared to NP-GPVI (LS: 29.9 ± 6.12 µm^2^; HS: 39.69 ± 13.16 µm^2^), NP-GPVI-CD39 significantly reduced mean thrombus size at LS by 30%. The overall thrombus count within a defined area was not different at LS. However, the overall thrombus count was significantly reduced by 16% (1640 ± 454) for NP-GPVI-CD39 at HS compared to NP-BSA (1955 ± 521). To summarize, both functional NPs reduced thrombus formation compared to control samples (Figure 7c). In addition, the reduced thrombus area and mean size in NP-GPVI-CD39 compared to NP-GPV revealed an additive effect of the enzymatic subunit CD39 of the NP-GPVI-CD39 construct. The reduced mean thrombus size may indicate a local inhibition of platelet-ADP-mediated platelet activation.

Following quantitative and planimetric analysis of the gained flow chamber images, additional intensity surface plots based on the fluorescence intensity levels of the data sets were prepared (Figure 7d,e). These data give an indication about the size and height of thrombi, as the fluorescence intensity increases when the fluorescent platelets accumulate during thrombus formation. Similar to the above-described statistical analysis, the resulting intensity surface plots showed smaller thrombi in the NP-GPVI and NP-GPVI-CD39 samples compared to the NP-BSA control. Topographical, three-dimensional models generated by scanning ion-conductance microscopy (SICM) visually reflect the statistical results (Figure 7f). Fewer thrombi were seen on a NP-GPVI-CD39 pre-incubated cover slip after perfusion when compared to NP-BSA. Moreover, thrombi appeared to be higher within NP-BSA samples (see color scale). Both analyses confirmed the results of the traditional flow chamber analysis.

**Figure 8:**
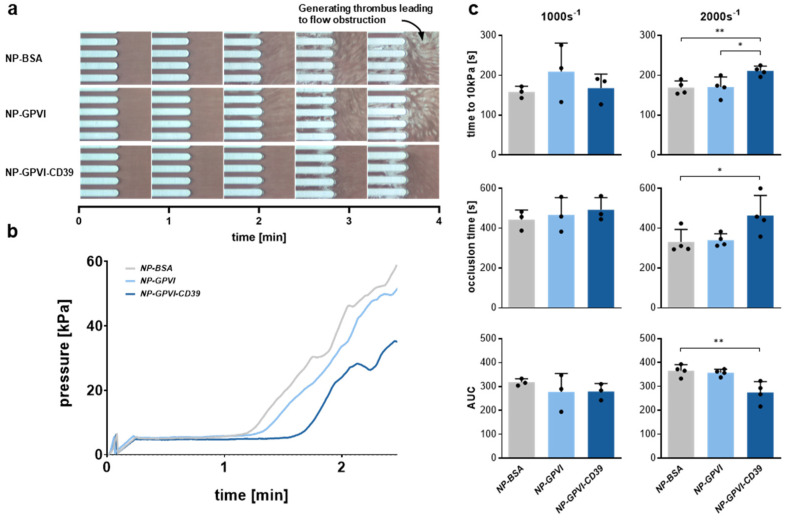
Receptor-coated nanoparticles interfere with thrombus formation under variable flow conditions. (**a**) Time series displaying progressive thrombus formation and consequent blood flow obstruction of the nanoparticle sample at a high shear rate (2000 s^−1^) through an automated total thrombus-formation analysis system (T-TAS^®^) assay. (**b**) Representative graphical comparison of pressure–time curves of the nanoparticle sample at high shear rates (2000 s^−1^). (**c**) Statistical analyses of the occlusion start time (time to reach 10 kPa), occlusion time (time to reach 60 kPa) and area under the curve (AUC) at both low (1000 s^−1^) and high (2000 s^−1^) shear rates. Mean ± SD; 1000 s^−1^: *n* = 3, 2000 s^−1^: *n* = 4; one-way ANOVA; * *p* ≤ 0.05, ** *p* ≤ 0.01.

Furthermore, the influence of the receptor-coated nanoparticles on in vitro thrombus formation was examined through an automated total thrombus-formation analysis system (T-TAS^®^, Fujimori Kogy Co Ld, Shinjuku, Japan) experiment under variable flow conditions (Figure 8). At high shear rates, NP-GPVI-CD39 significantly prolonged occlusion start time (time to 10 kPa [s]) by 25% (OST: 211.0 ± 12.19 s) compared to NP-GPVI (OST: 170.5 ± 25.42 s) and NP-BSA (OST: 169.3 ± 16.92) (Figure 8b,c). NP-GPVI-CD39 significantly prolonged occlusion time (time to 60 kPa [s]) at high shear rates by 40% (OT: 464.3 ± 100.5 s) in comparison to NP-BSA (OT: 331.3 ± 62.3 s). Furthermore, NP-GPVI-CD39 pre-incubation significantly decreased by 25% (275.2 ± 45.2) the AUC in comparison to NP-BSA samples. No significant differences were observed between samples at low shear rates. Therefore, these results further proved the inhibitory effect and the additive effect of CD39 of NP-GPVI-CD39, especially in a whole blood environment.

## 3. Discussion

Nanotechnology revolutionized various scientific fields [14]. Nanomedicine is still a very young field in medicine and is applying cutting-edge bioscientific technologies. Nanoparticle flexibility, in regard to modification of biochemical properties, surpasses traditional pharmaceuticals in pharmacodynamics and kinetics, biodegradability, immunogenicity and toxicity [15]. Nanoparticles have been successfully used in cancer therapy to achieve a highly localized application of drugs with low general toxicity. Even though CVD are a predominant cause of death worldwide, so far this field has not been extensively studied in regard to nanomedicine. Especially when, as in this case, the therapeutic nanoparticles could be easily applied and delivered directly through the vascular system. In this regard, conducting fundamental research on functionalized nanoparticles that can interact with exposed subendothelial collagen, which is a trigger for arterial atherosclerotic plaque formation, could lead to the development of new treatment options.

For this reason, PLGA nanoparticles were covered with either BSA, dimeric GPVI-Fc or GPVI-CD39 to create functionalized nanotherapeutics. As Ungerer et al. and Degen et al. have shown efficacy of both dimeric GPVI-Fc and fusion protein GPVI-CD39 in soluble form [7,8,13], further research needed to be conducted, comparing soluble versus nanoparticle-bound GPVI-Fc and GPVI-CD39 in terms of pharmacodynamic and kinetic properties. Levi et al. already observed effective collagen-binding capacity of NP-GPVI in in vitro and in vivo experiments [20]. Additionally, specific targeting of cerebral aneurysms via NP-GPVI had been verified [21].

The morphological characterization of NP-BSA, NP-GPVI and NP-GPVI-CD39 revealed general similarity regarding their diameter and height as well as their fluorescence properties, as verified by electron microscopy, AFM and flow cytometry. Electron microscopy revealed coating differences between the BSA control particles and the NP-GPVI and NP-GPVI-CD39 nanoparticle samples. NP-GPVI and NP-GPVI-CD39 were visible as a distinct electron-dense halo around the nanoparticle core, whereas BSA particles do not show this phenotype.

The in vitro influence of the functional nanoparticles NP-GPVI and NP-GPVI-CD39 on platelet aggregation and thrombus formation has been examined through a variety of assays. Enhanced collagen-binding capacity of NP-GPVI and NP-GPVI-CD39 was observed under both static and dynamic conditions. NP-GPVI-CD39 showed an augmented collagen-binding capability in comparison to NP-GPVI. As both nanoparticle formulations express an identical collagen receptor, GPVI-Fc, with CD39 being inert towards collagen, manufacturing deviations and GPVI-Fc expression variations may be considered as a possible explanation. Furthermore, higher coating densities may lead to receptor dysfunction due to increased proximity of the receptors to each other, thereby interfering with collagen adhesion properties. Moreover, excessive coating densities may promote off-target adhesion [22]. Further optimization of the particles should include adjusting the coating density of GPVI-CD39. Higher loading may be obtained by various methods; however, the coating density needs to be optimized such that off-target adhesion and effects remain low.

An inhibitory impact of NP-GPVI and NP-GPVI-CD39 on the aggregation process was confirmed following collagen and CRP activation by light transmission aggregometry. The new NP-GPVI-CD39 construct proved to be the only nanoparticle capable of significantly reducing the aggregation process following ADP-activation. The pre-incubation of ADP with NP-GPVI-CD39 nanoparticles lead to ADP degradation and inactivation through the ectonucleotidase CD39. Furthermore, the enzymatic activity of CD39 within the assay locally disrupted the ADP-activation cascade induced by the secretion of platelet-ADP, which otherwise has a multiplier effect. Thrombus formation interference was confirmed by flow chamber experiments, simulating physiological conditions. NP-GPVI and NP-GPVI-CD39 proved to effectively reduce thrombus coverage and mean thrombus size under low and high shear rates. In comparison to NP-GPVI, NP-GPVI-CD39 augmented the inhibition of in vitro thrombus formation significantly, constituting its bifunctional inhibitory impact on both platelet aggregation and thrombus formation. This observation was verified through T-TAS^®^ experiments performed with whole blood where NP-GPVI-CD39 showed significant interference with thrombus formation under high-shear conditions compared to both NP-GPVI and NP-BSA samples. As dysfunctional endothelium and subendothelial collagen exposure are mainly located in high-flow areas such as arterial or stenotic vessels, these findings suggest a potentially high interference of NP-GPVI-CD39 with thrombus formation specifically at those sites. As all conducted experiments were in vitro assays, in vivo functional assessment of the receptor-coated nanoparticles must be examined in future research projects.

There are limitations to this study. Only one batch of nanoparticles was analyzed. Even though soluble derivates of the coating have been analyzed we do not have a direct comparison of NP-bound constructs to soluble proteins. This could provide additional information about the advantages of NP delivery compared to soluble proteins. In addition, in vivo studies would be favorable and should be attempted next.

To conclude, in the present study, we generated functional nanoparticles coupled to recombinant fusion proteins that can bind to collagen (GPVI-Fc) and are linked to the ecto-ADPase CD39 (GPVI-CD39), which degrades pro-thrombotic extracellular ADP. We found that GPVI-CD39-coupled nanoparticles exhibited an enhanced antithrombotic activity in in vitro assays compared to GPVI-Fc-coupled nanoparticles. Our findings imply that bifunctional fusion proteins, in combination with the nanoparticle technology, are an attractive strategy for site-directed antithrombotic therapy.

## 4. Materials and Methods

### 4.1. Nanoparticle Generation and Characterization

The polymeric nanoparticles were prepared by the oil-in-water emulsion–solvent evaporation method. Briefly, PLGA (Acid terminated, lactide: glycilide—50:50, *M*_W_ 24.000–38.000, Sigma-Aldrich) and fluorescent dye (Nile red, Sigma-Aldrich, St. Louis, MO, USA) were dissolved in Dichloromethane (Bio-Lab Ltd., Jerusalem, Israel) and transferred dropwise to a 1% polyvinyl alcohol (PVA, 87–90% hydrolyzed, *M*_W_ 30.000–70.000, Sigma-Aldrich, St. Louis, MO, USA) solution. The solution was then emulsified in ice water by a probe sonicator (Vibra-Cell™ Ultrasonic, Sonics & Materials, Inc., Newtown, Connecticut, USA) followed by solvent evaporation.

BSA, GPVI-Fc and GPVI-CD39 were conjugated to the nanoparticles via NHS-EDC chemistry. EDC-NHS coupling is widely used for protein coupling [23,24] and has been implemented previously for PLGA NP-GPVI [20], whereas measurements we performed on non-covalent absorption showed minimal coupling to the surface. Average coating densities on the NPs were estimated by measuring the remaining protein after bioconjugation, performed via a standard Bradford assay - Pierce™ Coomassie Plus (TS-23236, Thermo Scientific, Waltham, MA, USA) [24,25]. Briefly, to estimate the number of protein molecules per particle, first, the unbound protein concentration was measured in the supernatant using a standard Bradford assay. It was then subtracted from the initial concentration to evaluate the amount of protein that remained on the particles. By dividing the amount of protein by the estimated number of particles, the number of protein molecules per particle was evaluated.

PLGA particle size distribution was determined by dynamic light scattering (Zetasizer Nano-ZS, Malvern Instruments, Malvern, UK) at 25 °C with a scattering angle of 173°. Particle surface charge (Zeta-potential) was measured using laser Doppler micro-electrophoresis (Zetasizer Nano-ZS, Malvern Instruments, Malvern, UK). For the final preparation stage, the particles were reacted in a blocking buffer (50 mM Tris, pH 8.0, 0.5% (*w/v*) casein) for 2 h at room temperature. The particles were washed again and preserved in the blocking buffer at 4 °C. The nanoparticles were suspended in Dulbecco’s phosphate buffered saline (PBS) (Sigma-Aldrich, St. Louis, MO, USA) at a stock concentration of 5 mg/mL and stored at −20 °C.

Primary morphological assessment of the nanoparticles was achieved through negative staining, ultra-thin sectioning, electron microscopy (Libra 120, Zeiss, Jena, Germany) and confocal laser scanning microscopy (LSM 800, Zeiss, Jena, Germany). Quantitative analysis of morphology parameters was executed by atomic force measurements (MFP3D-BIO Asylum Research, Santa Barbara, CA, USA) on NP-BSAs. Cantilever D (k = 5 pN/nm) by Brucker AFM Samples (MLCT) with a scanning rate of 0.1 Hz was employed to image nanoparticles on collagen-coated cover slips. Data analysis was performed with the open-source SPM software Gwyddion (http://gwyddion.net/; accessed on 2 March 2021).

### 4.2. Flow Cytometry

Flow cytometric measurements were executed with FACSLyric^TM^ (BD Biosiences, San Jose, CA, USA). The nanoparticles were resuspended in 0.22 µm filtered PBS at a target concentration of 5 µg/mL. In addition, a suspension containing 1 µm polystyrene beads was analyzed (F-13838, Thermo Fisher, Waltham, MA, USA) as per the manufacturer’s instructions. Forward and side scatter detection (FSC; SSC) delivered information about nanoparticle size and granularity, respectively. Through an upstream 585/40 nm band-pass filter (Phycoerythrin-channel) and a 670 nm long-pass filter (Peridinin-Chlorophyll-Protein-channel), fluorescence analysis of resuspended particles was achieved after sample excitation with a 488 nm laser. FlowJo Version 10.5.3 (BD Biosciences, San Jose, CA, USA) was employed for data visualization.

### 4.3. Fluorescent Microscopy and Flow Chamber

Static collagen adhesion capacity was observed through fluorescent/DIC microscopy using a Nikon Eclipse Ti2-A (Nikon Instruments Europe BV, Amsterdam, The Netherlands) under oil immersion. Nanoparticle suspensions (500 µg/mL) were incubated on collagen-coated cover slips (25 µg/mL) for 1 h at 37 °C. Unspecific molecular binding to collagen was prevented by pre-incubation with 1% BSA in phosphate buffered saline (PBS). PBS was used to wash off unbound nanoparticles before imaging. Quantitative image analysis was carried out using NIS-Elements AR (Version 5.21.00, Nikon Instruments Europe BV, Amsterdam, The Netherlands) and ImageJ (Version 1.52, National Institutes of Health, Bethesda, MD, USA). Fluorescent area, nanoparticle count and the average number of adhesive nanoparticles per 50 µm of collagen fiber were determined. Dynamic collagen binding was analyzed by flow chamber experiments. The nanoparticles were resuspended in PBS at 250 µg/mL. Consequent perfusion was carried out with a FlowChamberB.V. (Maastricht, The Netherlands) flow chamber at low (1.000 s^−1^; 7.53 mL/h) and high (1.700 s^−1^; 12.75 mL/h) shear rates. Real-time imaging of nanoparticle adhesion was achieved by fluorescence microscopy using a Nikon Eclipse Ti2-A microscope (Nikon Instruments Europe BV, Amsterdam, The Netherlands). Quantitative analysis was carried out by the NIS-Elements AR analysis software (Version 5.21.00, Nikon Instruments Europe BV, Amsterdam, The Netherlands).

Further flow chamber experiments were conducted with a FlowChamber B.V. flow chamber at LS and HS rates to examine thrombus formation interference. Cover slips were coated with a 100 µg/mL collagen suspension (collagen reagent HORM^®^, Takeda Austria GmbH, Linz, Austria). Next, the nanoparticles (200 µL; 5 µg/mL) were adhered to the cover slips for 1 h at room temperature. A 1% BSA solution was applied to inhibit unspecific bindings. Samples of 800 µL of anticoagulated whole blood from healthy donors were diluted with 200 µL of PBS. Ten min before perfusion, the fluorescent dye, DiOC6, was added to the blood samples. During the perfusion, platelets interacted with the collagen-coated cover slips, resulting in platelet activation and thrombus formation. Fluorescence microscopy with a Nikon Eclipse Ti2-A (Nikon Instruments Europe BV, Amsterdam, The Netherlands) enabled the observation of real-time thrombus formation. Afterwards, the chamber was perfused with PBS to wash off non-adhesive residue. Images of five independently selected areas were taken after the perfusion. Quantitative analysis and data transformation was carried out by NIS-Elements AR (Version 5.21.00, Nikon Instruments Europe BV, Amsterdam, The Netherlands).

### 4.4. Aggregometry

In order to assess the nanoparticles’ influence on platelet aggregation, light transmission aggregometry experiments with a CHRONO-LOG Aggregometer 490-X (Chrono-log Corporation, Havertown, Pennsylvania, PA, USA) were conducted. Platelet-rich plasma (PRP, platelet count: 100 × 10^6^) and platelet-poor plasma (PPP) from healthy donors were prepared by an established centrifugation protocol. The platelet aggregation activators ADP (5 µM), Collagen (2 µg/mL) and CRP (0.125 µg/mL) were pre-incubated with the nanoparticles (respective nanoparticle concentrations per agonist: ADP: 5 µg/mL; Collagen: 20 µg/mL; CRP: 20 µg/mL) for 30 min at 37 °C with the addition of CaCl_2_ (2 mM) to augment CD39 enzymatic activity. The aggregation activator sample (20 µL) was then added to PRP. Consequent aggregation activation was measured for 5 min against a baseline PPP sample. The measured parameters were maximal aggregation and AUC.

### 4.5. Scanning Ion Conductance Microscopy

SICM measurements were executed with a nanopipette (*r*_i_ ~ 50 nm; P-2000; Sutter Instrument, Novato, CA, USA) to scan and topographically remodel generated thrombi on the examined cover slips with a scanning area of 100 × 100 μm^2^. Thrombus morphology analysis was carried out by IGOR PRO 8 (WaveMetrics, Inc., Tigard, OR, USA).

### 4.6. T-TAS

In order to assess nanoparticle-induced interference on in vitro thrombus formation after whole-blood pre-incubation, automated total thrombus-formation analysis system (T-TAS^®^, Fujimori Kogyo Co. Ltd., Tokyo, Japan) experiments were conducted. Using PL-Chips for T-TAS^®^, whole blood was perfused through 26 collagen-coated microcapillaries and arranged in parallel under physiological arterial shear stress (low shear: 1.000 s^−1^; high shear: 2.000 s^−1^). Prior to perfusion, nanoparticles (20 µg/mL) were added to anti-coagulated whole-blood from healthy donors. Based on flow pressure measurements, thrombus formation interference was derived: The higher the flow pressure levels, the more pronounced the thrombus formation process, producing a pressure–time curve. The measured parameters were occlusion start time (time to reach 10 kPa), occlusion time (time to reach 60 kPa) and the area under the curve (AUC).

### 4.7. Statistics

General statistical analyses and graphical data visualizations were carried out by GraphPad Prism (Version 8.4.0, GraphPad Software, San Diego, CA, USA). Both ordinary and repeated measures one-way ANOVAs with Turkey’s or Dunnett’s multiple comparison tests were employed adequately for statistical significance testing with *p* ≤ 0.05 indicating statistical significance. Data are presented and given as means ± SD.

## Data Availability

Not applicable.

## Supplementary Materials

The following are available online at https://www.mdpi.com/article/10.3390/ijms23010011/s1.
