# Supplementary material for: Characterization of GPVI- or GPVI-CD39-Coated Nanoparticles and Their Impact on In Vitro Thrombus Formation
Source: Int J Mol Sci. 2021 Dec 21;23(1):11. doi: 10.3390/ijms23010011 (PMC8744670; doi:10.3390/ijms23010011)
Supplement: Supplementary file 1 [file ijms-23-00011-s001.zip › ijms-1487230-supplementary.pdf]

Supplemental Figure S1

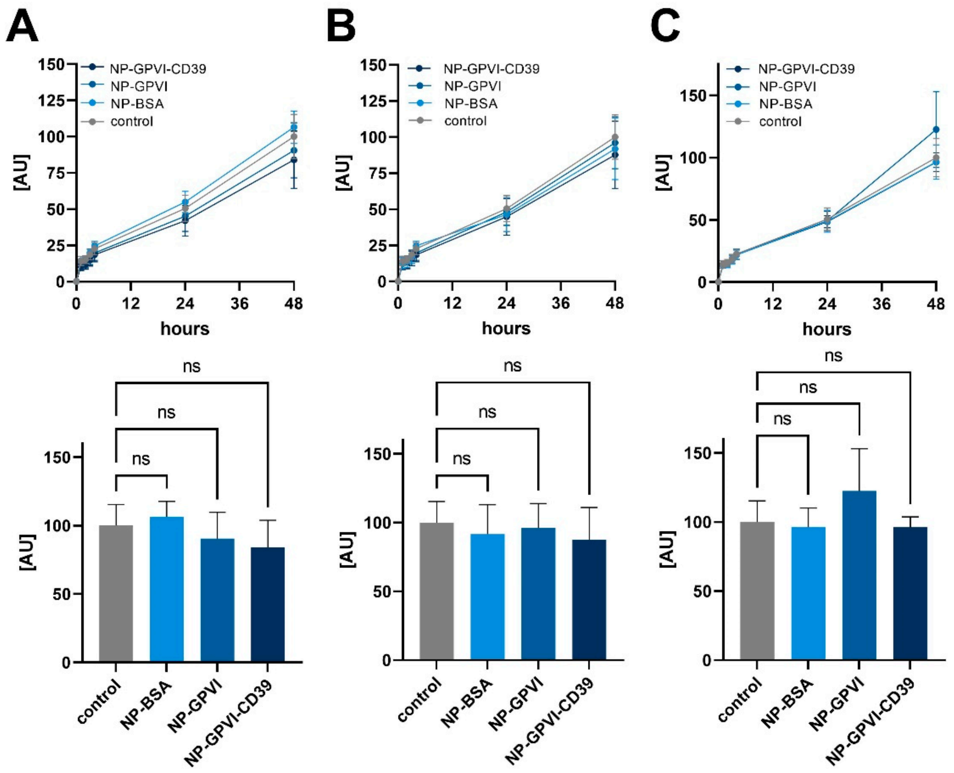

Supplemental Figure S1: RealTime-Glo™ MT Cell Viability Assay performed with **A** 5 µg/ml, **B** 20 µg/ml or **C** 50 µg/ml did show normal viability in all treated samples compared to the untreated control over 48 h (**upper row**). No significant differences were observed after 48 h incubation (**lower row**). Plotted: Mean±S.E.M.; n=3; Statistic: one-way ANOVA; n.s. = not significant.
